# Effectiveness of a Lytic Phage SRG1 against Vancomycin-Resistant* Enterococcus faecalis* in Compost and Soil

**DOI:** 10.1155/2017/9351017

**Published:** 2017-09-24

**Authors:** Sidra Rahmat Ullah, Saadia Andleeb, Taskeen Raza, Muhsin Jamal, Khalid Mehmood

**Affiliations:** ^1^Department of Industrial Biotechnology, Atta-ur-Rahman School of Applied Biosciences, National University of Sciences and Technology, Islamabad, Pakistan; ^2^Department of Microbiology, Abdul Wali Khan University, Garden Campus, Mardan, Pakistan; ^3^Department of Pharmacy, Abbottabad University of Sciences & Technology, Hazara, Pakistan; ^4^Department of Pharmaceutics, College of Pharmacy, University of Hail, Hail, Saudi Arabia

## Abstract

Nosocomial infections caused by vancomycin-resistant* Enterococcus* have become a major problem. Bacteriophage therapy is proposed as a potential alternative therapy. Bacteriophages are viruses that infect bacteria and are ubiquitous in nature. Lytic bacteriophage was isolated from sewage water that infects VREF, the causative agent of endocarditis, bacteraemia, and urinary tract infections (UTIs). The phage produced clear plaques with unique clear morphology and well-defined boundaries. TEM results of phage revealed it to be 108 ± 0.2 nm long and 90 ± 0.5 nm wide. The characterization of bacteriophage revealed that infection process of phage was calcium and magnesium dependent and phage titers were highest under optimum conditions for VREF, with an optimal temperature range of 37–50°C. The maximum growth was observed at 37°C, hence having 100% viability. The latent period for phage was small with a burst size of 512 viral particles per bacterial cell. The phage was tested against various clinical strains and results proved it to be host specific. It can be used as a potential therapeutic agent for VREF infections. The phage efficiently eradicated VREF inoculated in cattle compost, poultry compost, and a soil sample which makes it a potential agent for clearing compost and soil sample.

## 1. Introduction

*Enterococcus faecalis* is a facultative anaerobe, nonmotile, Gram-positive bacterium found in soil, water, food, plants, and sewage [1]. Enterococci inhabit oral cavity, intestines, and female genital tract of human and are recognized as opportunistic pathogens. Enterococci not only are the causative agents of UTIs, bacteraemia, and endocarditis but also infect the bloodstream, intra-abdominal and pelvic regions, surgical sites, and central nervous system [2]. The occurrence of vancomycin-resistant enterococci (VRE) has reached endemic magnitude in various medical centers due to antibiotic resistance [3]. This resistance can be intrinsic, as against most beta-lactams and aminoglycosides [4], or acquired due to either mutations in the genome or addition of resistance encoding genes, as against tetracycline, macrolides, chloramphenicol, and glycopeptides [5]. VRE pose a serious medical threat leaving no treatment option for some infections on one hand and transfer of such resistance traits to other opportunistic pathogens on the other [3, 6].

Bacteriophages are emerging as potential biocontrol agents that specifically target some bacteria in both animals and humans [7]. Today bacteriophages are considered as potential option to be used as antimicrobial agents due to increasing failure of antibiotics. Phages were used for the first time in 1919 as therapeutic agents [8]. Phage therapy is therapeutic use of bacteriophage to destroy pathogenic bacteria [9]. Phage therapy has been proven to be superior to conventional chemotherapy in certain cases [10, 11]. In recent years increasing focus on phage control has been observed as diseases caused by multiple drug-resistant bacteria, including VRE, are becoming more prevalent [12].

Effective tools must be adopted to ensure VRE-free animal and agriculture environment. Avoparcin, growth promoter and homologue of vancomycin, is extensively used in animal husbandry and agricultural industry [13]. Nontherapeutic use of antibiotics in agriculture leads to antibiotic resistance in gut bacteria, such as* Enterococci*. Not only can this resistance infect people, but these resistance genes can be transferred to other organisms (World Health Organization 2003). Animal production is in close relation to human society due to the use of animal products and recycling resources. The risk of VRE contamination in human population can easily be increased with incidence of its contamination in animal husbandry. As animal waste compost is widely used in farming, it should be ensured that compost is VRE-free to avoid its contamination in agriculture land [12].

Among 36 phages previously isolated for* Enterococcus* [14], only few against VRE have been reported [9, 12, 15, 16]. The objective of this study was to isolate and characterize bacteriophage against vancomycin-resistant* Enterococcus faecalis* as a potential agent for VRE control. The aims of the study included the bacterial reduction in natural as well as inoculated compost and soil.

## 2. Materials and Methods

### 2.1. Bacterial Identification

After overnight incubation of bacterial strain, established microbiological methods (colony morphology, Gram staining, and biochemical test) were used for identification of bacterial strain [1]. Analytical profile index (API) test kit [11], a standardized identification system for Enterococcaceae family, was used for biochemical tests. Kirby Bauer method was used to check the antimicrobial susceptibility of the strain. For 16S rRNA sequencing, DNA was manually isolated using methods described by Carozzi et al., 1991. The 16S rRNA gene was amplified by using polymerase chain reaction (PCR) with universal 16S rRNA primers (RS-1, 5′-AAACTCAAATGAATTGACGG-3′; RS-3, 5′-ACGGGCGGTGTGTAC-3′) as described earlier [8]. Reaction was carried out by initial denaturation at 95°C for 5 minutes and 35 cycles of amplification (95°C: 45 seconds, 51°C: 45 seconds, and 72°C: 1 minute) with a final elongation time of 5 minutes at 72°C. The amplified product was electrophoresed on 1% agarose gel and eluted from gel using Invitrogen gel extraction kit (Carlsbad, USA). Sequencing was carried out by Molecular Biology Products (Karachi, Pakistan). The sequence homology was checked by subjecting the 16S rRNA sequence to BLAST analysis (https://www.ncbi.nlm.nih.gov/BLAST).

### 2.2. Phage Enrichment and Amplification

Bacteriophages specific to VREF were enriched with methods described by Stenholm et al. with some modifications [17, 18]. Sewage water sample was centrifuged at 1400 rpm for 15 minutes to remove algal cell and debris. Sewage water sample was incubated with isolated host bacterial strain to enrich the phage population.

The above prepared sample concentrates (5 mL) were added to 30 mL OD_600_ VREF grown overnight at 37°C. Enrichment cultures were incubated overnight at 37°C with shaking at 150 rpm. Enrichment culture was incubated overnight and 1 mL was transferred to eppendorf tubes and one drop of 1% chloroform was added to disrupt bacterial cells and release phages. The culture was centrifuged at 14000 rpm for 10 minutes to pellet down bacterial cell debris. The supernatant was filtered using 0.45 *μ*m and 0.20 *μ*m syringe filters sequentially (Minisart filters, Germany) and transferred to a new tube. Plaque assay on LB agar plate (tryptone 10 g/L, yeast extract 5 g/L, NaCl 10 g/L, and agar 15 g/L) with top agar (LB media having 0.75% agar) was carried out for the detection of phage. Autoclaved test tubes with 100 *μ*L of sewage sample along with 100 *μ*L of the VREF culture were incubated for 5–10 minutes at room temperature. About 2-3 mL of top agar was added to the test tubes and the tubes were poured to appropriate plates. The plates were then allowed to solidify after which they were incubated at 37°C overnight and examined for plaques. SM buffer [100 mM NaCl, 8 mM MgSO4, and 50 mM Tris HCl (pH 7.5)] and 0.002% (w/v) gelatin at 4°C with the addition of 7% dimethyl sulfur oxide (DMSO) were used to store the purified phages at –80°C.

### 2.3. Calcium and Magnesium Ions Effect on Adsorption Rate of Phage

A culture of VREF (100 mL), incubated at 37°C for 16 h, was taken and divided into four flasks of 25 mL each. Among two sets of flasks, one was inoculated with 500 *μ*L (10 × 10^7^ pfu/ml) phage only (control), while the second flask was inoculated with both 500 *μ*l phage and 250 *μ*l CaCl_2_ (10 mM). In the other set, one flask was inoculated with 500 *μ*L (10 × 10^7^ pfu/ml) phage only (control), while the second flask was inoculated with both 500 *μ*l phage and 250 *μ*l MgCl_2_ (10 mM). Samples were taken at different time intervals of 0, 10, 20, and 30 minutes to measure the number of free phages in control and Ca and Mg added solution. The number of free phages was determined using the double layer agar assay [18].

### 2.4. One-Step Growth, Latent Period, and Phage Burst Size

The method previously described for determining the latent period and burst size by Jamal et al. (2015) was followed [18]. VREF culture (50 mL) was incubated to mid exponential phase O.D_600_ of 0.4–0.6 and the cells were harvested by centrifugation. Pellet was resuspended in 0.5 mL LB broth media and mixed with 0.5 mL phage having 10 × 10^7^ pfu. Phage was allowed to adsorb to bacteria for 1 minute and mixture was centrifuged at 13000 rpm for 30 seconds to remove free phages. The pellet was resuspended in 100 mL fresh media and culture was incubated at 37°C continuously. Samples from the incubated flask were taken at 3-minute intervals and the phage titer was determined by double layer agar assay.

### 2.5. Transmission Electron Microscopy of SRG1 Phage Morphology

The purified SRG1 phage particles were transferred to the surface of a formvar carbon film (on a 200-mesh copper grid), negatively stained by addition of 2% uranyl acetate, and blotted immediately with filter paper; the grid was then air-dried for viewing by transmission electron microscopy (Hitachi, H-7000) at 100 kV. The guidelines provided by international committee on taxonomy of viruses (ICTV) were used for classification of the SRG1 phage based on the electron microscopy results.

### 2.6. Thermal and pH Stability

The pH stability for phage was carried out as previously described by Capra et al. [19, 20]. Under pH values of 1, 3, 5, 7, 9, and 11, 500 *μ*L of phages was treated and incubated at 37°C for 1 hour. After incubation, each treated sample was tested against the host in double layer agar assay to check the viability of phages.

Thermal stability test for the phage was performed according to the method described by Capra et al. [19, 20]. Phage filtrates were taken in eppendorf tubes and treated under different temperatures for 1 hour. After incubation, plaque assay was performed for each treated sample.

### 2.7. Host Range Determination

The host specificity of the phage was assessed on a range of clinical pathogenic bacterial strains including* Pseudomonas aeruginosa*, Methicillin resistant* Staphylococcus aureus* (MRSA),* Citrobacter* sp.,* Acinetobacter* sp.,* E. coli, E*.* faecalis*, and* E*.* faecium*. To test the susceptibility of bacterial isolates, drop-on-lawn technique was used [21]. After overnight incubation at 37°C, plates were checked for any plaque formation against an uninfected negative control.

### 2.8. Bacterial Reduction Assay

Two flasks containing 100 mL LB broth media were inoculated with VREF and incubated at 37°C in shaking incubator. When O.D_600_ was in the range of 0.4–0.6, one flask was inoculated with 1 mL of phage filtrate. The other flask was taken as control having no phages. The flasks were then incubated at 37°C and the O.D_600_ readings were taken at interval of 2 hours for 24 hours using spectrophotometer [18].

### 2.9. Application of Bacteriophage to Compost and Soil

Compost is organic matter that has been decomposed and recycled as a fertilizer and is a key ingredient in organic farming. In LB media, 7 ml of bacterial culture was grown and CFU (colony forming unit) was calculated (3.5 × 10^9^). The poultry and cattle compost samples along with soil sample were measured and dried at 70°C for 4 hrs. 1.5 mL of bacterial culture was taken in an eppendorf and was then centrifuged at 13,000 rpm for 10 minutes. Supernatant was removed and pellet was washed twice with double distilled water.* E*.* faecalis *along with double distilled water was enumerated in LB media. 1 mL of diluted* E. faecalis* (3.2 × 10^7^) was added to 0.5 g of both compost and soil samples. The compost and soil sample were added to each flask and 500 *μ*l of phage filtrate was added. The mixture was vortexed and incubated at 37°C for 2 days. Soil and compost were diluted with 5 mL of sterilized dH_2_O and vortexed for 2 minutes. Serial dilutions were made and bacterial colonies were enumerated on LB agar plates [12].

## 3. Results

### 3.1. Bacterial Identification

**Figure 1:**
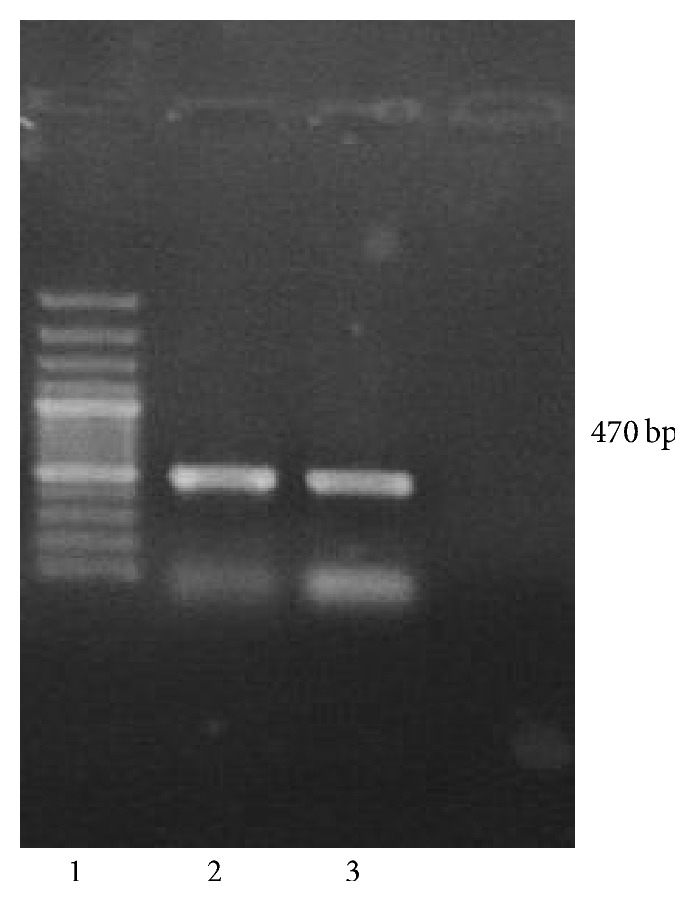
PCR amplification of* Enterococcus faecalis* 16S rRNA gene. Lane 2 and lane 3 show bands at approximately 470 bp. Lane 1 shows 1 kb ladder.

Morphological characterization showed off white/beige colored round pinpoint colonies with entire margins.* Enterococcus* was confirmed to be Gram-positive cocci by using Gram staining technique. The strain was confirmed to be VREF which belongs to Enterococcaceae family by biochemical test API 20E. PCR yielded an expected size amplicon (470 bp) that was subjected to DNA sequencing from both orientations (Figure 1). The resulting sequence was deposited to database and aligned to search for homologous sequences. In Basic Local Alignment Search Tool (BLAST) analysis, it showed high nucleotide sequence identity of 100% to* Enterococcus faecalis*. The sequence was submitted to NCBI and accession number was obtained as* Enterococcus faecalis* KF448071.

The Kirby Bauer method showed that the strain was resistant to antibiotics including vancomycin, erythromycin, teicoplanin, and ciprofloxacin.

### 3.2. Bacteriophage Isolation

**Figure 2:**
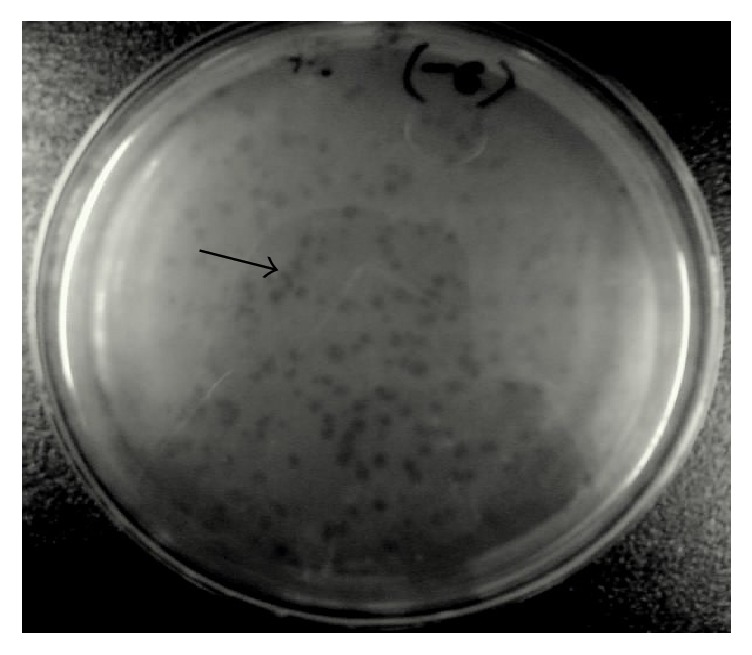
Double layer agar assay, arrows showing pinpoint plaques of phage. Calculated pfu was 10 × 10^7^ with plaque size of 0.2–0.5 mm in diameter.

Phage SRG1 was isolated for VREF from water sample collected from sewage in Islamabad, using the double layer agar assay technique after enrichment of the phage. Plaques were obtained after incubation of plates at 37°C overnight having plaque size ranging from 0.2 to 0.5 mm in diameter and unique clear morphology with well-defined boundaries (Figure 2).

### 3.3. Analysis of Calcium and Magnesium Ions Effect on Phage Adsorption Rate

**Figure 3:**
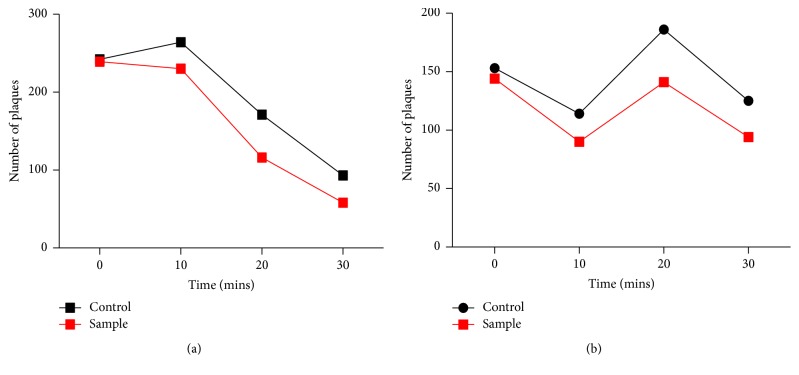
(a) Effect of calcium ions on adsorption rate of phage and VREF. (b) Effect of magnesium ions on adsorption rate of phage and VREF. Divalent metal ions had positive effect on the binding of phage to bacterial cell surface receptors as evident by decreased number of free phages in comparison to controls.

The effect of calcium and magnesium ions on adsorption of phage was analyzed by adding (10 mM) CaCl_2_ and MgCl_2_ to the phage and VREF. The number of free phages left in the solution at different time intervals of 0, 10, 20, and 30 minutes was detected using the plaque assay. Statistical analysis showed significant difference between control and the calcium ion treated group (Figures 3(a) and 3(b)). The ions might have enhanced phage adsorption.

The percentile of free phages was found by the following formula:

(1) $$\begin{array}{c} \text{Percentage of free phage}=\left(\;\frac{N}{N_{o}}\;\right)\times 100, \end{array}$$

where *N*_*o*_ is the PFU/ml of phages at *T* = 0 min while *N* is PFU/ml at *T* = 10, 20, and 30 mins.

### 3.4. Latent Period and Phage Burst Size

**Figure 4:**
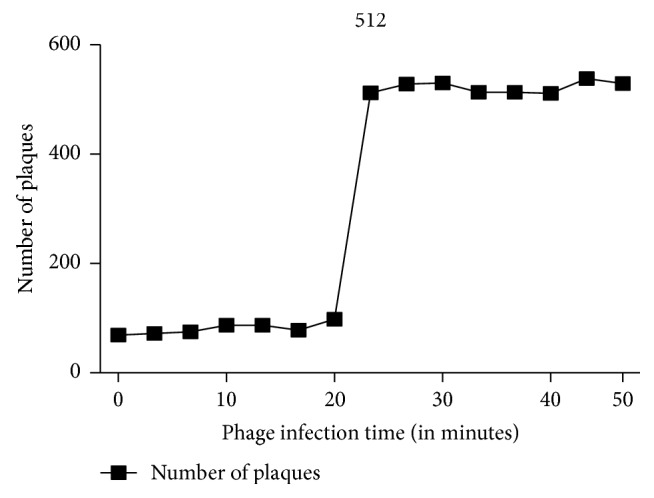
One-step growth experiment showing latent phase (21 mins) with burst size (amount of infectious virus produced, per infected cell) of 521 phages and plateau and of the phage SRG1.

One-step growth experiment was performed for determination of the latent time period and burst size of phage. The graph obtained shows the lag, log, and the stationary phase (Figure 4). The latent time was determined to be 21 minutes. The burst size of the phage was 512 virions. Determination of burst size was based on the ratio of mean yield of phage that infected the bacterial cells to the mean phage particles liberated.

### 3.5. Transmission Electron Microscopy of SRG1 Phage Morphology

**Figure 5:**
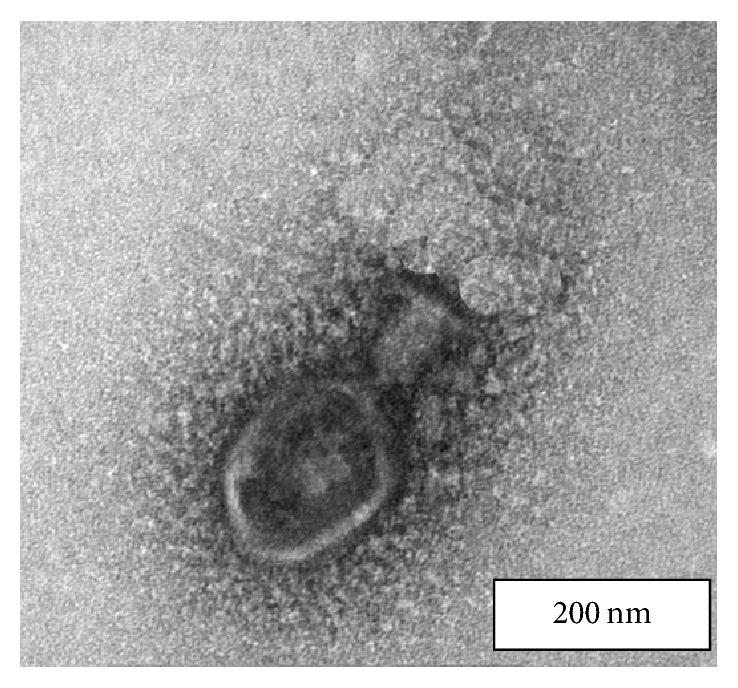
Transmission electron micrographs of the purified SRG1 phage; scale bars, 200 nm.

The phage morphology was observed by transmission electron microscopy. The phage head was 108 ± 0.2 nm long and 90 ± 0.5 nm wide with icosahedral sides and a long tail of about 107 ± 03 nm and width 29 nm. The phage was assigned to family Myoviridae on the basis of its morphology (Figure 5).

### 3.6. pH and Thermal Stability

**Figure 6:**
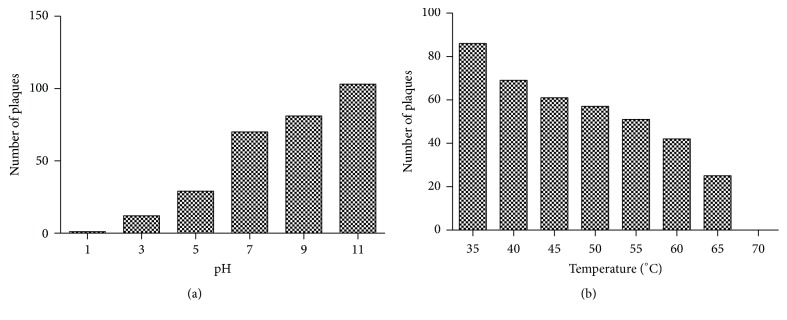
(a) pH stability test showed maximum infectivity at extreme basic pH. (b) Heat stability test showed that 37°C is optimum temperature for phage SRG1.

Phage stability was checked in a range of pH in order to determine optimum pH for phage. At pH 7, incubation for one hour showed no apparent reduction. Number of phages reduced at extreme pH 3.0 and pH 5.0 whereas no active phage was found at pH 1. The number of phages increases with increasing pH. (Figure 6(a)).

The potential of phage to resist heat was checked by heat stability test. At 37°C, phage showed 100% activity and found to be stable till 37–65°C. No phages could be observed at 70°C (Figure 6(b)).

### 3.7. Host Range Determination

Clinical strains of eight pathogens including* Pseudomonas aeruginosa*, Methicillin resistant* Staphylococcus aureus* (MRSA),* Citrobacter* sp.,* Acinetobacter *sp.*, E. coli, E*.* faecalis*, and* E*.* faecium* were used to determine the host range of phage by means of drop-on-lawn technique [21]. The plates were incubated overnight at 37°C. The phage showed slight activity against* E. faecalis* but overall it had a narrow host range (Table 1).

### 3.8. Bacterial Reduction Assay

**Figure 7:**
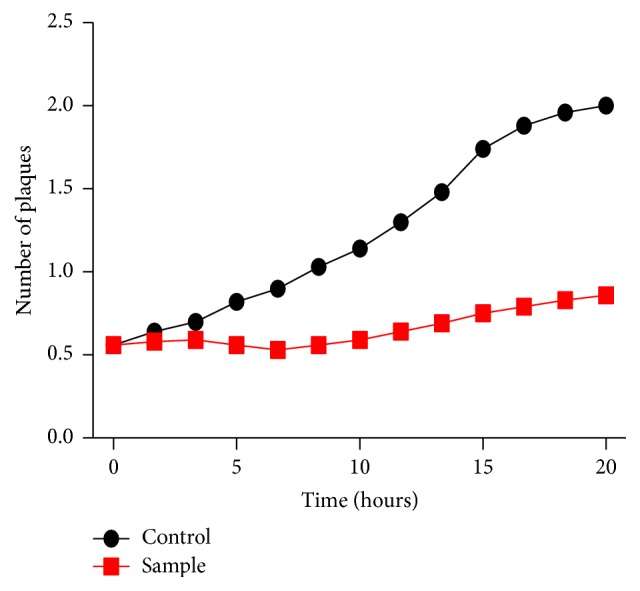
Lysis of VREF in broth culture by phage SRG1 over the time period of 20 hrs.

Infection of VREF was observed with phage for 24 hrs. The turbidity of VREF broth decreased remarkably due to phage infection as compared to the control (Figure 7). The comparison of O.D_600_ for phage infected and control culture revealed remarkable difference. However, after 11 hours of incubation increase in O.D_600_ was observed which might be attributed to phage resistant bacterial cells or the cell debris (Figure 7).

### 3.9. Bacterial Reduction in Compost

Three types of cattle compost, 1 poultry compost, and soil samples were inoculated by the* E. faecalis*. Bacteriophage was added and the bacterial reduction was observed after 24 and 48 hours and compared with controls without phage. The compost and soil samples were measured and dried at 70°C for 4 hrs. About 1 mL of diluted VREF broth was added to 0.5 g of both compost and soil samples. 500 *μ*l of phage filtrate was added to each flask and after incubation CFU was calculated (Table 2).

## 4. Discussions

*Enterococcus* species are known to be opportunistic organisms causing fatal and difficult-to-treat infections [1]. The emergence of resistance in* Enterococci* against antibiotics of aminoglycoside, *β* lactams, and glycopeptides (vancomycin and teicoplanin) is a major problem for medical community. Vancomycin and teicoplanin are the agents of choice for infections caused by enterococci [4] and emergence of VRE poses a serious threat because of increased mortality and morbidity [22]. The prevalence of VRE has increased dramatically and six VRE outbreaks have been reported in literature to date, worldwide [23–28].

Bacteriophage therapy can serve as alternate for VRE infections as contemporary medicine has failed to cope with this serious problem. In countries like Russia, Poland, and Eastern Europe bacteriophage therapy has been used for enteric and systemic treatments [8]. Institute of Bacteriophage, Microbiology and Virology, Georgia, is a center for phage therapy and conducts research programs to use phage as a priority for bacterial infections [8].

In this study, we have isolated lytic phage named SRG1 specific for clinical isolate of vancomycin*-*resistant* Enterococcus faecalis* (VREF) from sewage water [17]. Other groups have also reported isolation of phages against VRE [9, 12, 15] from sewage, compost, and water channel. Temperature, pH, metal ions CaCl_2_ and MgCl_2_, and the physiological stage of host are among the factors that affect in vitro activity of the phage [29, 30]. According to previous reports metal ions may be involved in the adsorption, attachment, and penetration of phage in the host cell [31, 32]. Ca and Mg ions have been suggested to stabilize the weak interaction of phage with receptors by increasing the phage population on host surface that increases the alteration of cell surface receptors in insertion of phage genome into the host cell [33, 34]. Phage SRG1 showed more infectivity with a 10 mM calcium chloride and magnesium chloride concentration.

In this study, phage showed increased activity at pH range of 7–11. The reason may be adaptation of phage to slightly basic pH of sewage water from which it has been isolated. In our study, the phage showed excellent stability to alkaline pH and its infectivity reduced at acidic pH. The optimum temperature for phage was observed to be 37°C and this can be explained as* Enterococcus* being part of normal flora of humans. Adsorption rate of phage as well as the metabolic activities of host was elevated by increase in temperature [32].

One-step growth experiments exhibited all stages of phage life cycle. Latent time period of the phage was found to be 21 minutes and studies report optimum latent time attributes to high phage fitness [35]. The gradual rise can be attributed to cell debris as well as the phage resistant cells that have developed immunity against the phage [36].

Host range of SRG 1 was checked on a range of clinical pathogenic bacterial strains and the results showed that the phage was highly host specific. The infectivity of phage to some isolates of* E. faecalis* was observed with spot assay; however, in plaque assay no phage activity was observed. The reason for infectivity in spot assay might be the cytolysin produced by the bacteria.

Application of phage 10^7^ pfu/ml to three different cattle composts and one poultry raw compost having 10^8^ cfu/ml showed complete eradication of not only experimentally inoculated bacteria but also naturally occurring* E. faecalis*. 50% bacterial reduction was observed in experimentally inoculated soil sample. In a study by Otawa, the phage vrep-5 led to the decrease of VRE abundance up to >3 log_10_ [12]. In consideration of today's need for organic recycling of animal waste, troubles with pathogens and bacterial drug resistance need to be resolved by inexpensive and effective methods. Enormous amounts of animal waste are emitted daily, and as yet, we have no efficient way of killing specific pathogens in compost. Our study provides a considerable way of killing these pathogens present in compost and soil by means of phage particles.

## 5. Conclusions

Bacteriophage isolation against VRE is a major step for the development of strategies to control VRE infections. Phage characterization shows that it belongs to family Myoviridae which not only efficiently lyses VRE but also has outstanding thermal and pH stability that makes it a potential candidate for antibacterial therapy. However, the phage is highly host specific and is not effective against other clinical isolates included in our study which suggests that more virulent phages are yet to be discovered in the future. The phage completely eradicated experimentally inoculated VRE in compost and soil samples which proves it to be a potential agent for treatment processes.

## Figures and Tables

**Table 1:** Determination of host range of phage by using spot and plaque assay.

Sr number	Hospital strain number	Bacterial strain	Bacteriophage effect	
			Spot assay	Plaque assay
(1)	2934	*Citrobacter *sp.	(—)	(—)
(2)	2938	*Staphylococcus aureus*	(—)	(—)
(3)	3568	*Pseudomonas aeruginosa Z*	(—)	(—)
(4)	2895	*Staphylococcus aureus*	(—)	(—)
(5)	2966	*Acinetobacter *sp.	(—)	(—)
(6)	3168	*E. coli*	(—)	(—)
(7)	2908/22	*E. coli*	(—)	(—)
(8)	2953/5	*E. coli*	(—)	(—)
(9)	U-806	*E. faecalis*	Activity	(—)
(10)	TH-785	*E. faecalis*	(—)	(—)
(11)	B-215	*E. faecalis*	(—)	(—)
(12)	FT-490	*E. faecalis*	(—)	(—)
(13)	H-707	*E. faecalis*	(—)	(—)
(14)	U-439	*E. faecalis*	(—)	(—)
(15)	B-216	*E. faecalis*	Activity	(—)
(16)	FT-167	*E. faecalis*	(—)	(—)
(17)	U-170	*E. faecalis*	Activity	(—)
(18)	U-790	*E. faecium*	(—)	(—)
(19)	FT-607	*E. faecium*	(—)	(—)
(20)	FT-549	*E. faecium*	(—)	(—)

**Table 2:** The reduction of *E. faecalis* by phage in different samples; the phage titer pfu/ml 10 × 10^7^ was used along with cfu/ml of original culture 3.5 × 10^9^ and cfu/ml of diluted culture 1.6 × 10^8^.

Sample	CFU after 24 hrs, control	CFU after 48 hrs, control	CFU after 24 hrs, sample	CFU after 48 hrs, sample
Compost A (Plantfert)	1.95 × 10^8^	2.20 × 10^8^	0	0
Compost B (few)	2.04 × 10^8^	2.25 × 10^8^	0	0
Compost C (Plantafor, Germany)	1.74 × 10^8^	1.86 × 10^8^	0	0
Compost P	1.8 × 10^8^	2.16 × 10^8^	7.0 × 10^7^	2.4 × 10^7^
Soil sample	1.88 × 10^8^	2.11 × 10^8^	9.0 × 10^7^	1.10 × 10^7^
